# Diffusion-weighted imaging diagnostic algorithm in patients with suspected pleural malignancy

**DOI:** 10.1007/s00330-021-08013-6

**Published:** 2021-05-28

**Authors:** Wenrui Jiang, Zhiping Han, Xing Tang, Hong Yin, Jian Zhang

**Affiliations:** 1 Department of Respiratory Medicine, Xijing Hospital, Fourth Military Medical University, No. 127 Changle West Road, Xi’an, 710032 China; 2 Department of Radiology, Xijing Hospital, Fourth Military Medical University, Xi’an, China

**Keywords:** Diffusion magnetic resonance imaging, Pleural neoplasms, Tomography, X-ray computed

## Abstract

**Objectives:**

The purpose of this study was to analyze the diagnostic performance and clinical application of diffusion-weighted imaging (DWI) in patients with suspected pleural malignancy (PM).

**Methods:**

A retrospective review of patients with suspected PM was performed from March 2014 to August 2018 (NCT 02320617). All patients underwent chest DWI and computed tomography (CT) with cytological or histopathological findings as reference standards. The diagnostic performance of DWI and CT was analyzed and compared. A DWI diagnostic algorithm with three sequential steps was established.

**Results:**

Seventy patients (61.6 ± 13.6 years; 47 males and 23 females) were included. The sensitivity of DWI (94.2%, 49/52) for the diagnosis of PM was significantly higher compared with CT (67.3%, 35/52), with similar specificity (72.2% vs. 72.2%, respectively). The apparent diffusion coefficient of malignant lesions (1.15 ± 0.32 × 10^−3^ mm^2^/s) was lower compared with benign lesions (1.46 ± 0.68 × 10^−3^ mm^2^/s), but the cutoff value was difficult to define for overlap between groups. Approximately 62.5% (5/8) of invasive procedures were avoided when using the DWI diagnostic algorithm in patients with suspected PM without N3 lymph node or extra-thoracic metastasis.

**Conclusion:**

Including DWI into the diagnostic algorithm of suspected PM can effectively identify malignancy and avoid unnecessary invasive procedures, which may have some potential in clinical application.

**Key Points:**

• *Diffusion-weighted imaging can identify pleural malignancy much more efficiently than CT.*

• *A diffusion-weighted imaging diagnostic algorithm helped to avoid unnecessary invasive procedures in patients without N3 lymph node or extra-thoracic lesions.*

• *A hyperintense signal on DWI at a high b value (800 s/mm*^*2*^*) but not at a low b value (50 s/mm*^*2*^*) was a reliable signature of PM.*

**Supplementary Information:**

The online version contains supplementary material available at 10.1007/s00330-021-08013-6.

## Introduction

Identifying pleural malignancy (PM) is vital, since it signifies advanced cancer and poor prognosis [1–4]. Chest computed tomography (CT) is the most widely used modality in the diagnosis of PM [5–7]. However, pleural dissemination can be observed in 1.2–4.6% of patients who undergo surgery [8–10], since a negative report from a routine scan is insufficient to rule out malignancy [11–13]. Thus, an additional invasive procedure is often necessary [4, 14, 15].

Magnetic resonance imaging (MRI) was previously used to diagnose pleural disease, but the technique demonstrated poor spatial resolution caused by movement artifacts from the lung and heart [14]. After decades of progress, image quality has greatly improved [16–19].

Diffusion-weighted imaging (DWI), which is a functional analysis that can exploit the random movement of water molecules in tissues and indirectly reflect the increased cellularity resulting from tumors, can differentiate PM from benign lesions [20]. However, most studies use either a complex apparent diffusion coefficient (ADC) calculation, which greatly depends on the subjective evaluation of the radiologist [21, 22], or a prolonged scan time with multiple *b* values [23]. These requirements restrict the utility of DWI in clinical practice. Thus, the present study aimed to retrospectively analyze the diagnostic performance of DWI and explore the clinical utility of DWI in patients with suspected PM.

## Methods

### Patients

**Fig. 1:**
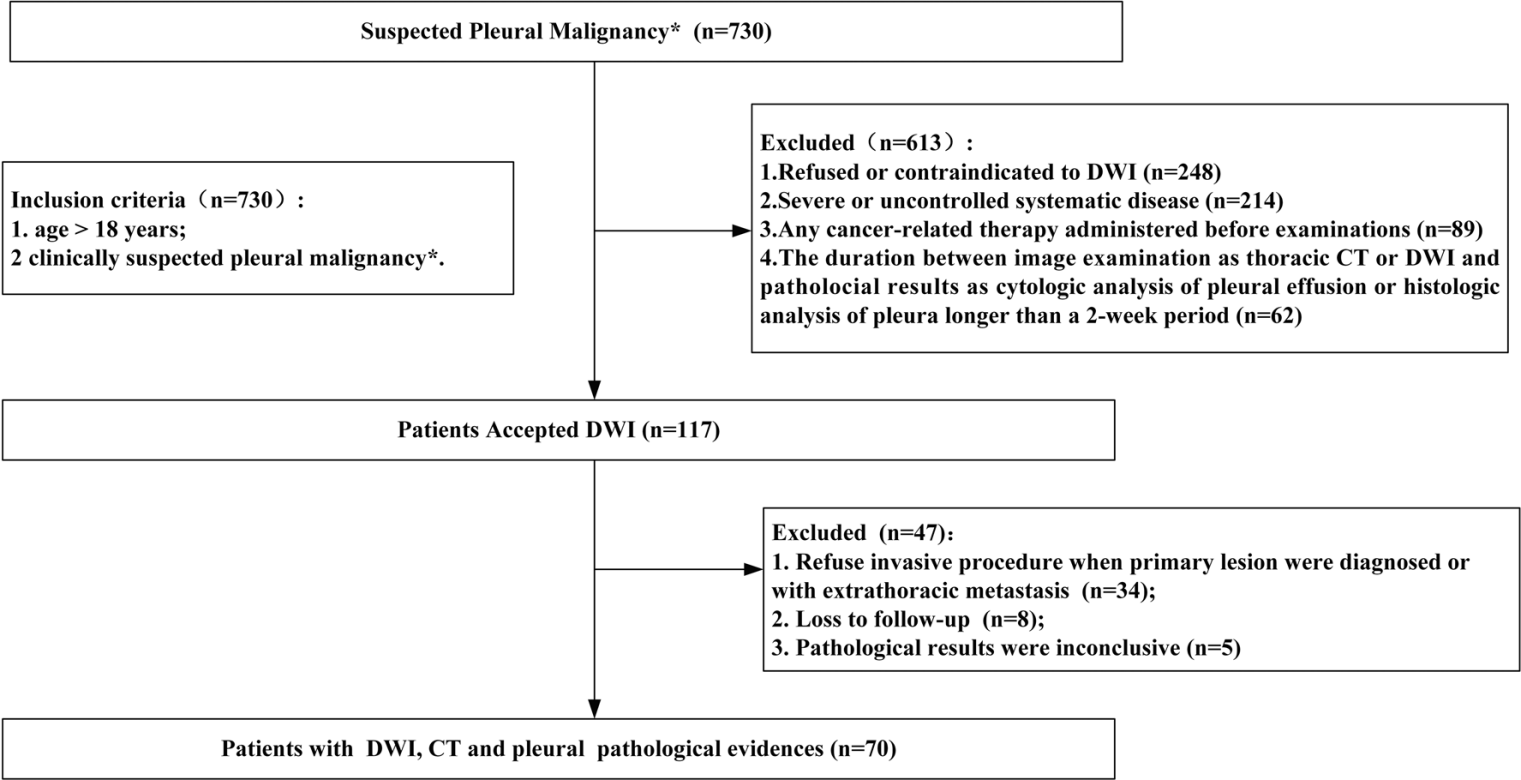
Flowchart of patient inclusion, exclusion, and recruitment. DWI: diffusion-weighted imaging. *Suspected pleural malignancy: (1) new effusion in patients with primary malignant disease; (2) any unexplained pleural lesion or effusion after primary care

A retrospective review of patients with suspected PM was carried out from March 2014 to August 2018 (NCT 02320617), and patient enrollment is summarized in Fig. 1. Since DWI is not commonly used for the diagnosis of pleural disease, patients enrolled in this study were mainly recruited from another prospective project (NCT 02320617), in which DWI was employed to analyze lung nodule. The inclusion criteria included (a) age > 18 years and (b) clinically suspected PM. Suspected PM was defined as new pleural effusion in patients with primary malignant disease or any unresolved pleural lesion or effusion after primary care. Seven hundred thirty patients met the criteria; however, a total of 613 patients were excluded mainly because DWI was not performed at the early stage of pleural evaluation: (a) refusal to undergo or contraindication to DWI; (b) severe or uncontrolled systemic disease; (c) cancer-related therapy administered before examination; (d) duration of thoracic CT, DWI, and cytological analysis of pleural effusion, or histological analysis of pleura, of > 2 weeks. A total of 117 patients underwent DWI. Thirty-four of these patients refused to undergo a further invasive procedure. Eight patients withdrew, and five patients had inconclusive pleural pathology results. Thus, a total of 70 patients were included. All chest CT and DWI scans were completed successfully and safely, and pathological verification was available for each patient. This retrospective study was approved by the institutional review board of Xijing Hospital, and written informed consent was obtained from all patients.

### Thoracic CT

The standard protocol included helical CT from the lung apices through the adrenal glands. The imaging parameters were as follows: 120 kVp; Care Dose 4D 60 mAs; rotation speed, 0.28 s; pitch, 1.0; slice thickness, 8 mm; reconstruction interval, 8 mm; lung parenchyma window level, −1500 HU; window width, 500 HU; mediastinum window level, 40 HU; window width, 300 HU.

### Chest DWI

Patients were in the supine position during the examination. MRI was performed using a 1.5-T superconducting magnet (Trio-Tim, SIEMENS Systems) using an eight-channel large flexible coil. The gradient field intensity was 40 mT, and the gradient switching rate was 150 T. Conventional axial T1-weighted (repetition time, 3.84 ms; echo time, 1.91 ms; 1 excitation) and axial T2-weighted (repetition time, 2200 ms; echo time, 86 ms; 1 excitation) sequences were performed during a suffocating state. Then DWI was performed during free breathing using single-shot spin-echo-plane imaging and parallel acquisition of a space-question sensitivity coding technique. The parameters were as follows: repetition time, 6800 ms; echo time, 83 ms; *b* values, 50 and 800 sec/mm^2^; field of view, 400 × 400 mm; matrix size, 156 × 156; slice thickness, 5 mm; section gap, 0 mm.

### Image analysis

DWI and CT images were analyzed by two radiologists with more than 10 years of experience who were both blinded to patients’ clinical information. A 5-point visual scoring system for both CT and DWI was used to assess the probability of malignancy on a per-site basis as follows: 1, definitely benign; 2, probably benign; 3, equivocal; 4, probably malignant; and 5, definitely malignant. Patients who scored 1, 2, or 3 were classified as benign, while patients who scored 4 or 5 were classified as malignant.

For CT analysis, we introduced another scoring system (CT^s^) as previously reported [13]. In simple terms, several representative features of PM on CT were scored (Supplemental Table 1). A sum score of ≥ 7 was used as the cutoff value for PM.

For DWI, after visual assessment, average ADCs were calculated for all pleural lesions in the corresponding region of interest (ROI) of the ADC map using a post-processing workstation (Syngo VE32B, SIEMENS Systems). The ROI was placed in the region with the most homogeneity and highest signal intensity on the DWI map, far from the lung–fluid interface and diaphragmatic regions to avoid magnetic susceptibility artifact.

### Diagnostic criteria

For each patient, at least three pleural effusion samples were sent for cytological analysis. Image-guided pleural biopsy or surgery was performed if needed. PM was diagnosed if malignant evidence was detected by pathologists with more than 25 years of experience. All patients classified as benign were followed up for a minimum duration of 6 months or until pleural effusion was completely resolved.

### Statistical analysis

This is retrospective diagnostic test. The main purpose was to compare the diagnostic performance (sensitivity, specificity, PPV, NPV, and accuracy) of DWI and CT for pleural malignancy. Approximately 150,000 patients were affected by PM in US [24]. To determine the sample size, we assumed the sensitivity and specificity of DWI in diagnosis of pleural malignancy are approximately 71.4 ~ 93.0% and 79.0 ~ 100%, respectively, according to the previous study [21, 23]. Then PASS (version 15.0) was used to calculate the sample size with tolerable error being 0.1 and α being 0.05. SPSS (version 19.0) and GraphPad Prism (version 6.01) were used for statistical analysis. Sensitivity, specificity, positive predictive value (PPV), negative predictive value (NPV), and accuracy of CT and DWI in the identification of PM were calculated according to the criteria described earlier. A *p* value of < 0.05 was considered statistically significant.

## Results

### Patient characteristics

**Fig. 2:**
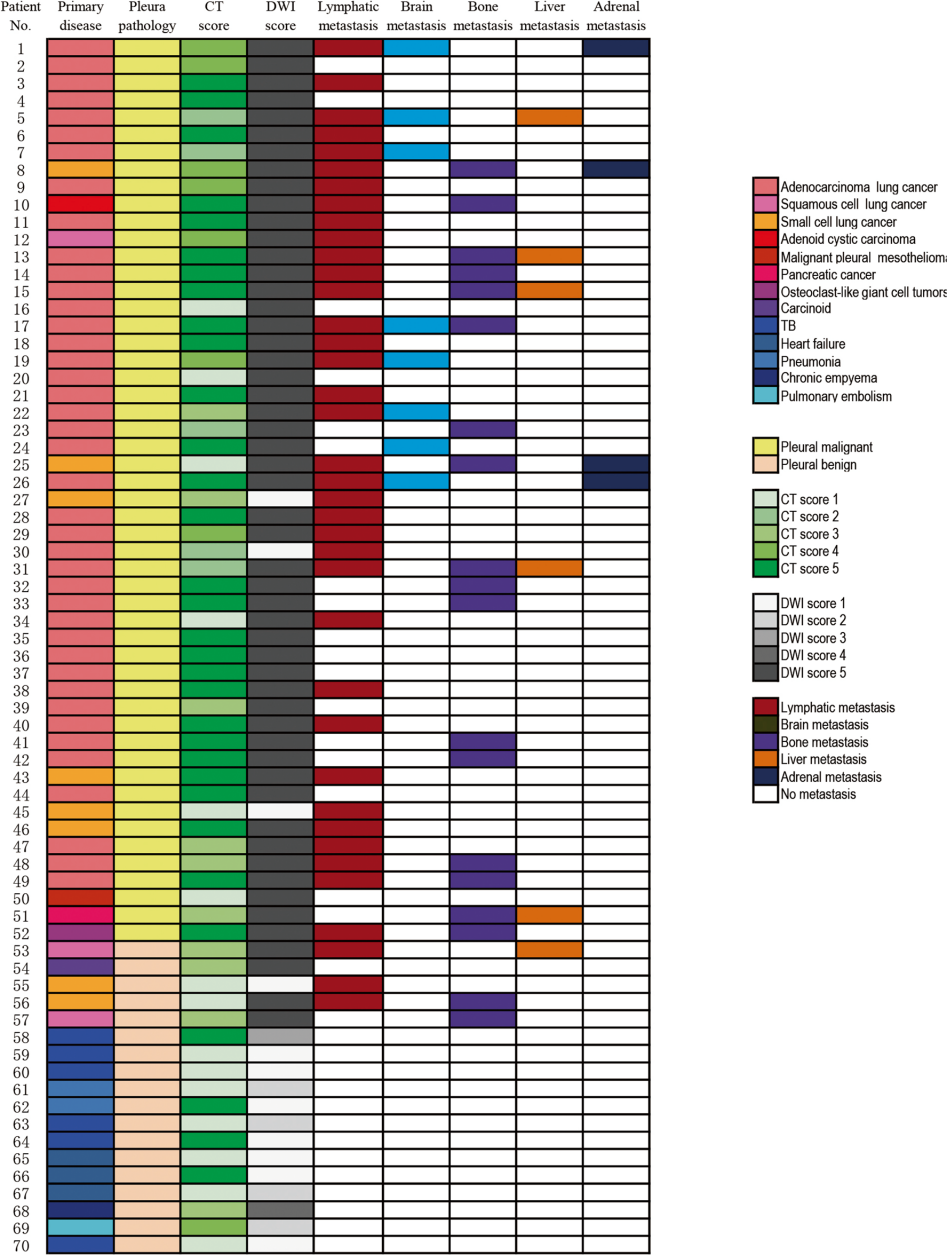
Patient number and diagnosis of primary disease, pleural pathology, visual score on CT and DWI, and metastasis in lymph nodes, brain, bone, liver, and adrenal glands. TB: tuberculosis

The baseline characteristics of 70 patients (47 males and 23 females) with a mean age of 61.6 ± 13.6 years (range, 20–88 years) are summarized in Table 1. The results of DWI, CT, pleural pathological diagnosis, and primary disease for each patient are demonstrated in Fig. 2. PM was diagnosed in 52 patients. Forty-nine patients had primary lung cancer, 41 of whom had adenocarcinoma, six of whom had small cell lung cancer (SCLC), one of whom had squamous cell carcinoma (SCC), and one of whom had adenoid cystic carcinoma. The other three patients had malignant pleural mesothelioma, pancreatic cancer, and osteoblast-like giant cell tumor with pleural dissemination, respectively. Eighteen patients were diagnosed with benign pleural lesions. For patient nos. 58, 59, 60, 63, 64, and 70, TB was diagnosed. For patient nos. 61 and 62, parapneumonic effusion was defined, as bacterial pneumonia was associated and pleural effusion was completely resolved after antibiotic therapy. For patient nos. 65, 66, and 67, heart failure was diagnosed and responded to diuretics. For patient no. 69, pulmonary embolism was diagnosed by CT-PA. Patient nos. 54 and 68 were diagnosed by surgery. Patient nos. 53, 55, 56, and 57 with primary tumors were closely monitored and pleural effusion was completely resolved in the 6-month follow-up.

**Table 1:** Baseline characteristics of patients

Patient characteristics	No.	%
Age (year)		
Average	61.6 ± 13.6	
Range	20–88	
Gender		
Male	47	67.1
Female	23	32.9
Smoking status		
Never	35	50.0
Former	19	27.1
Current	16	22.9
Pleural malignancy	52	74.3
Lung cancer	49	70.0
Adenocarcinoma	41	58.6
Small cell	6	8.6
Squamous cell	1	1.4
Adenoid cystic carcinoma	1	1.4
Malignant pleural mesothelioma	1	1.4
Pancreatic cancer	1	1.4
Osteoclast-like giant cell tumor	1	1.4
Pleural benign	18	25.7
Squamous cell lung cancer	2	2.9
Small cell lung cancer	2	2.9
Primary pulmonary carcinoid tumor	1	1.4
Tuberculosis	6	8.6
Heart failure	3	8.6
Pneumonia	2	2.9
Chronic empyema	1	1.4
Pulmonary embolism	1	1.4

### Chest CT image assessment

**Fig. 3:**
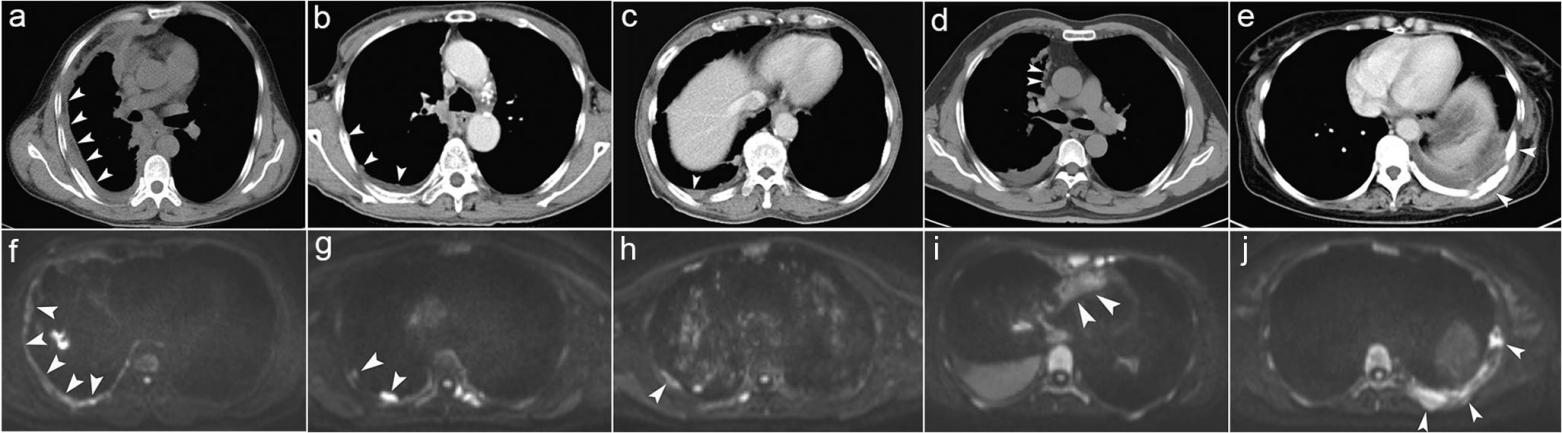
Representative pleural malignant features on CT (**a**–**e**) and DWI (**f**–**j**) are marked by the white arrow: **a** circumferential pleural thickening; **b** nodular pleural thickening; **c** visceral pleural thickening > 1 cm; **d** mediastinal pleural involvement; **e** chest wall invasion and rib destruction at multiple sites; **f** circumferential pleural thickening; **g** nodular pleural thickening; **h** thready pleural thickening; **i** mediastinal pleural involvement; **j** chest wall invasion and rib destruction at multiple sites

The representative features of PM on CT are shown in Fig. 3. The diagnostic performance of CT is shown in Table 2 (sensitivity, 67.3% [35/52]; specificity, 72.2% [13/18]; PPV, 87.5% [35/40]; NPV, 43.3% [13/30]; accuracy, 68.6% [48/70]). Pleural effusion is the only CT feature that may suggest benign pleural lesions. Nodular pleural thickening was observed in most patients in the PM group (61.50% [32/52]); however, none of the pleural features were significantly different between the malignant and benign groups (Supplemental Table 2).

**Table 2:** Qualitative assessment with DWI and CT to diagnose PM

Modality	Sensitivity (%) (95% CI)	Specificity (%) (95% CI)	PPV (%) (95% CI)	NPV (%) (95% CI)	Accuracy (%) (95% CI)
DWI	94.2 (49/52)	72.2 (13/18)	90.7 (49/54)	81.3 (13/16)	88.6 (62/70)
	(84.1–98.8)	(46.5–90.3)	(79.7–96.9)	(54.4–96.0)	(78.7–94.9)
CT	67.3* (35/52)	72.2 (13/18)	87.5 (35/40)	43.3 (13/30)	68.6 (48/70)
	(52.9–79.7)	(46.5–90.3)	(73.2–95.8)	(25.5–62.6)	(56.4–79.1)
CT^s^	82.7 (43/52)	61.1 (11/18)	86.0 (43/50)	55.0 (11/20)	77.1 (54/70)
	(69.7–91.8)	(35.7–82.7)	(73.3–94.2)	(31.5–76.9)	(65.6–86.3)

*CT* computed tomography, *CT*^*s*^ computed tomography scoring system, *DWI* diffusion-weighted imaging, *PM* pleural malignancy, *PPV* positive predictive value, *NPV* negative predictive value; *p = 0.0005 (CT vs. DWI)

The scoring results of CT^s^ are shown in Supplemental Table 3. The diagnostic performance of CT^s^ (sensitivity, 82.7% [43/52]; specificity, 61.1% [11/18]; PPV, 86.0% [43/50]; NPV, 55.0% [11/20]; accuracy, 77.1% [54/70]) was not significantly different when compared with the CT and DWI visual assessments (Table 2).

### DWI image assessment

As described in Table 2, 62 patients were correctly diagnosed by DWI (sensitivity, 94.2% [49/52]; specificity, 72.2% [13/18]; PPV, 90.7% [49/54]; NPV, 81.3% [13/16]; accuracy, 88.6% [62/70]) by visual assessment. The representative features of PM on DWI are also shown in Fig. 3. Hyperintense pleural areas on DWI, especially at a high *b* value (800 s/mm^2^) but not at a low *b* value (50 s/mm^2^), strongly suggested malignancy (Supplemental Table 4). This feature was present in 94.2% of patients (49/52). Other features, including circumferential pleural thickening, nodular pleural thickening, thready pleural thickening, mediastinal pleural involvement and chest wall invasion, and rib or centrum destruction, were not statistically different between the malignant and benign groups (Supplemental Table 4).

We also evaluated the performance of DWI in all patients without N3 lymph node or extra-thoracic metastasis. In total, 28 patients were analyzed (Supplemental Table 5). The sensitivity, specificity, PPV, NPV, and accuracy of DWI were 100.0% (13/13), 86.7% (13/15), 86.7% (13/15), 100% (13/13), and 92.9% (26/28), respectively. The respective CT values were 76.9% (10/13), 66.7% (10/13), 66.7% (10/15), 76.9% (10/13), and 71.4% (20/28).

The average ADC in the malignant group (1.15 ± 0.32 × 10^−3^ mm^2^/s) was lower compared with the benign group (1.46 ± 0.68 × 10^−3^ mm^2^/s), but no statistically significant difference was observed (Supplemental Figure 1). The ADCs of seven benign patients (38.8%, 7/18) and two malignant patients (3.8%, 2/52) were not measurable, since the pleura were not visible on DWI.

### DWI diagnostic algorithm

**Fig. 4:**
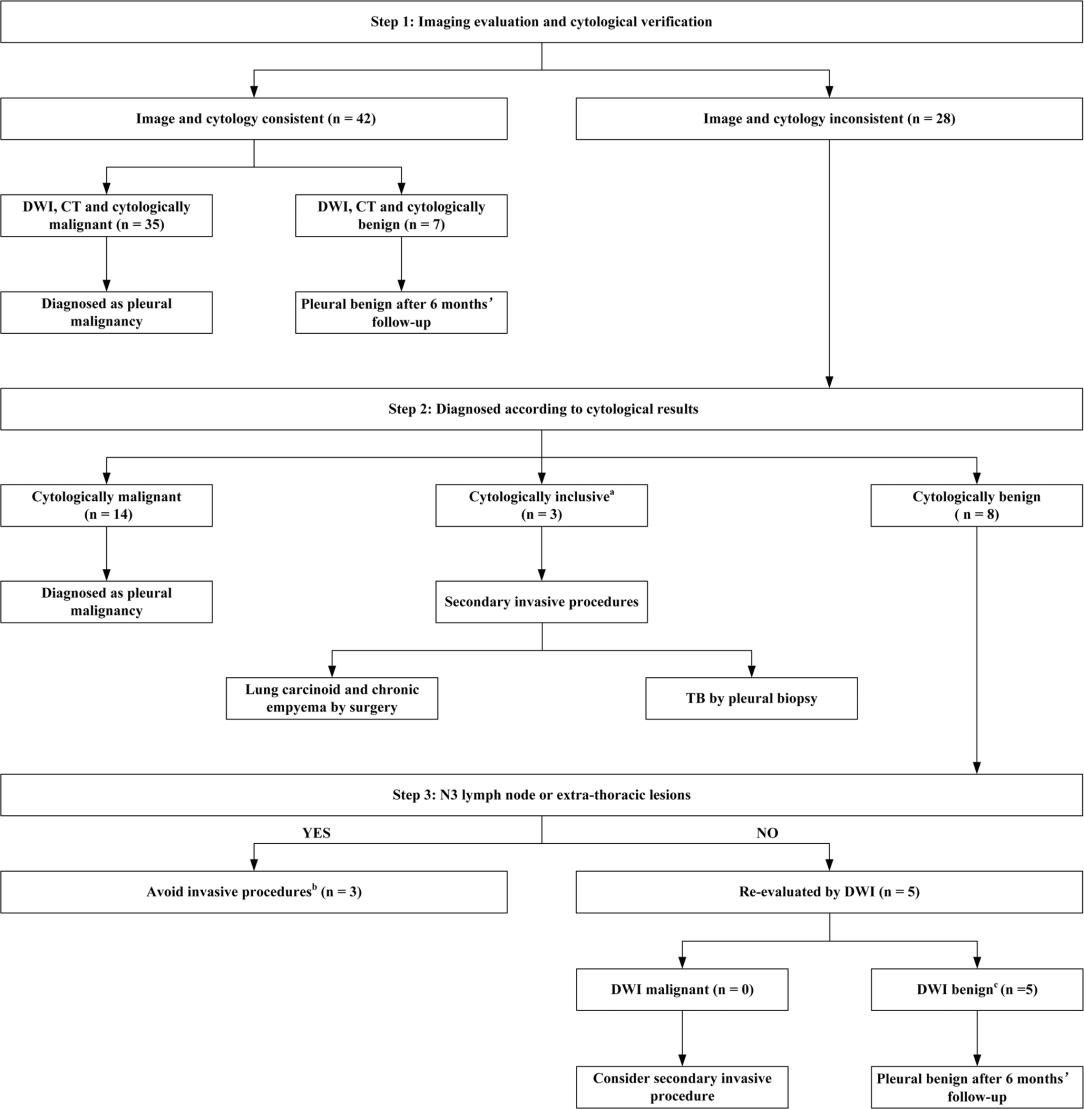
DWI diagnostic algorithm of suspected PM. (a) Cytological inclusion was defined as atypia cells in pleural effusion. (b) Invasive procedures were avoided in these patients because management would change marginally with the diagnosis of PM. (c) Although PM features on CT were observed in these patients, a benign diagnosis was supported by both DWI and cytological reports and confirmed after 6 months of follow-up. TB: tuberculosis; DWI: diffusion-weighted imaging; PM: pleural malignancy; CT: computed tomography

**Fig. 5:**
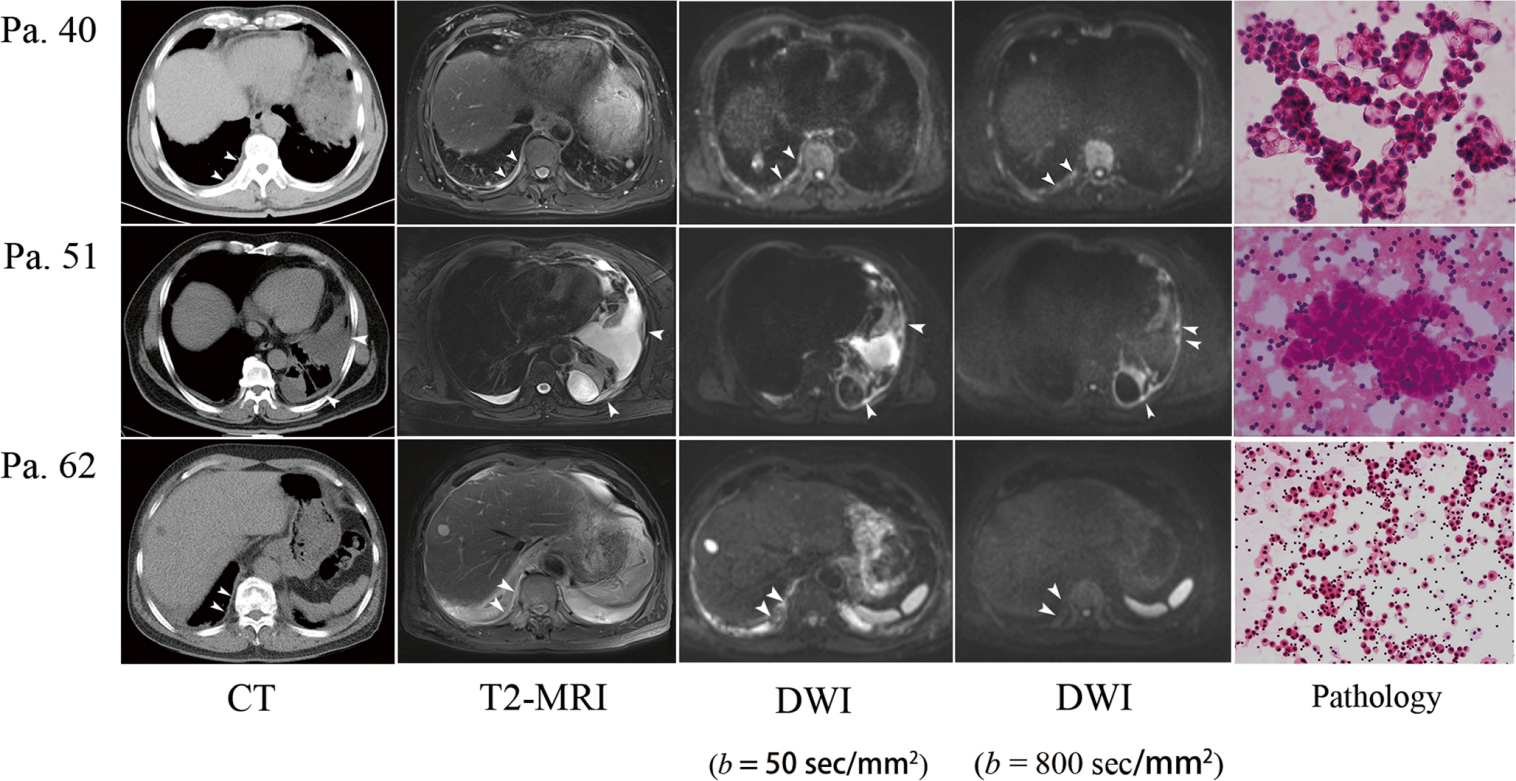
Representative cases. Patient 40 was a 60-year-old male diagnosed with primary adenocarcinoma. Circumferential pleural thickening was observed on CT and reported as PM. Similar features were observed on T2-weighted MRI. Multiple hyperintense areas were observed on DWI at a low *b* value (50 s/mm^2^) and a high *b* value (800 s/mm^2^), and definite malignancy was concluded. The cytological results confirmed adenocarcinoma. Patient 51 was a 63-year-old female diagnosed with advanced adenocarcinoma with bone and liver metastasis. CT was equivocal in the pleural assessment. Thready pleural thickening was observed on T2-weighted MRI. Multiple hyperintense areas were present on DWI, especially at a high *b* value (800 s/mm^2^), and definite malignancy was concluded. Cytological results confirmed adenocarcinoma. Patient 62 was a 67-year-old male with recurrent pleural effusion. Circumferential pleural thickening was observed on CT, and definite malignancy was concluded. However, hyperintense areas present on DWI at a low *b* value (50 s/mm^2^) were not present at a high *b* value (800 s/mm^2^); thus, the patient was classified as benign. Benign results were reported by cytology, which was repeated three times, and effusion was completely resolved after antibiotic therapy and 6 months of follow-up.

Both imaging and cytological results were considered when developing a diagnostic algorithm for patients with suspected PM (Fig. 4). In step 1, DWI, CT, and cytological results were consistent in 42 patients, and definitive diagnoses were made (Fig. 5). The other 28 patients with inconsistent imaging and cytological results were classified as cytologically malignant, inconclusive, or benign in step 2. Seventeen patients were diagnosed with PM with malignant pleural fluid cytopathic findings. A total of 82.35% of these patients (14/17) were recognized as having PM on DWI, but not on CT. Taking patient no. 51 as an example (Fig. 5), the CT report was equivocal, whereas malignant features (circumferential and multiple nodular pleural thickening) were clearly observed on DWI (Fig. 5), especially at a high *b* value (800 s/mm^2^). The other three patients with PM were recognized neither by DWI nor by CT. Inclusive cytological results (a few atypia cells were seen in pleural effusion) were reported in patients 54, 68, and 70; thus, surgery or pleural biopsy was performed.

The remaining eight patients with benign cytological results and malignant reports from either DWI or CT were managed in step 3. Presence of N3 lymph node or extra-thoracic metastasis was considered. Secondary invasive procedures were avoided in patients with late-stage cancer (patients 53, 56, and 57). For the other five patients with local lesions and malignant CT reports, further invasive procedures would normally have been performed; however, they were avoided in practice after DWI re-evaluation. In patient no. 62 (Fig. 5), circumferential pleural thickening was present on CT and DWI at a low *b* value (50 s/mm^2^), which is a typical malignant feature; however, circumferential pleural thickening was not observed on DWI at a high *b* value (800 s/mm^2^). Thus, PM was ruled out, and parapneumonic effusion was confirmed after 6 months of follow-up.

## Discussion

In this retrospective study, DWI outperformed CT in the diagnosis of suspected PM. Due to its high sensitivity, DWI recognized most patients with PM, while CT did not. Although the specificities of DWI and CT were similar, inclusion of DWI into the diagnostic algorithm helped to avoid unnecessary invasive procedures, especially in patients without N3 lymph node or extra-thoracic metastasis.

CT not being sufficiently sensitive to identify PM is becoming a consensus [12, 25], and this observation was confirmed in this study. Parietal pleural thickening (> 1 cm), nodular pleural thickening, mediastinal pleural thickening, and circumferential pleural thickening are representative features of PM on CT [26]; these features were first reported in 1990 and are still in use today. High sensitivities and specificities of these features have been reported in previous retrospective studies [5–7, 27, 28]. However, recent large-sample studies have re-evaluated the diagnostic performance of CT in pleural disease and reached different conclusions [11, 13, 25]. One study enrolled 370 patients with pleural effusion who underwent thoracoscopy after CT evaluation at two centers [11]. The sensitivity of CT was only 68%, and the specificity was 78%. Another study in 2017 enrolled 315 patients [25] and observed similar results with an overall sensitivity of 58% and a specificity of 80%. They also mentioned that the sensitivity of CT could be slightly increased to 68% by involving a specialized thoracic radiologist. These data are concordant with our study. CT^s^ [13] can be introduced to improve the diagnostic performance of CT. With CT^s^, Porcel et al reported high sensitivity (88%) and specificity (94%) values, which were also observed in our study (sensitivity, 82.7%; specificity, 61.1%). Differences between the results of Porcel et al and the results of the present study might be due to differences in the study population, area, and institution. Despite this, we have indicated the insufficiency of chest CT for the diagnosis of PM, irrespective of whether specialists are involved and a scoring system is used.

DWI has certain advantages for the diagnosis of malignant disease. First, T1-weighted images demonstrate excellent contrast in the presence of abnormalities in anatomical structure. Second, T2-weighted images can reveal tissue-specific information. Third, DWI is based on the mobility of water molecules within tissues, which can be quantified using the ADC. This information can be used to evaluate malignant lesions [16, 20, 29, 30]. In a previous study, we investigated the diagnostic capability of DWI for visual assessment of malignant pulmonary nodules [30]. We found that DWI had high sensitivity (96.1%), specificity (83.3%), and accuracy (92.0%). In the present study, we further retrospectively analyzed the diagnostic performance of DWI in patients with suspected PM. Normal parietal pleura is approximately 0.02 mm; thus, it is barely visible with DWI. We found that the hyperintense signal on DWI at a high *b* value (800 s/mm^2^) but not at a low *b* value (50 s/mm^2^) was a reliable signature of PM. This is similar to “pleural pointillism” reported in 2015, which has high diagnostic efficiency (sensitivity, 93%; specificity, 79%; accuracy, 88%) and is defined as multiple hyperintense pleural areas that gradually become visible at a low *b* value of 0 s/mm^2^ to high *b* values (50, 100, 500, 750, and 1000 s/mm^2^) [23]. However, scans performed using a series of different *b* values increase the scan time to several hours and reduce patient tolerance. In our study, the process was simplified by comparing images acquired using two settings. Thus, the scan time was shortened to approximately half an hour, and clinical utility and patient tolerance were improved significantly.

Whether the ADC can be used to evaluate PM is still controversial. In 2010 [20], a prospective study enrolled 62 patients with suspected malignant pleural mesothelioma. In this study, the ADC acquired from chest DWI was used as a biomarker to identify histopathological typing of MPM. Later in 2012, Coolen et al evaluated the performance of DWI in patients with malignant pleural disease and reported that the sensitivity, specificity, and accuracy were 71.4%, 100%, and 87.1%, respectively, with a cutoff ADC value of 1.52 × 10^−3^ mm^2^/s [21]. In 10 patients with an ADC of between 1.52 and 2.00^−3^ mm^2^/s, dynamic contrast-enhanced MRI further improved the sensitivity, specificity, and accuracy to 92.8%, 94.1%, and 93.5%, respectively. However, another study in 2016 observed great ADC overlap between malignant and benign pleural lesions [22]; thus, a sufficiently discriminative cutoff value could not be obtained. Similar results were shown in our study. Although there was a tendency for the ADC to be lower in malignant lesions compared with benign lesions, no statistically significant difference was observed. One major reason is that a large proportion of benign lesions was invisible on MRI; thus, the ADC could not be obtained. In addition, the ADC calculation depends on the ROI subjectively chosen by the radiologist and could thus introduce information bias. Application of the ADC in the diagnosis of pleural disease requires further exploration.

Proper management of suspected PM greatly depends on accurate image evaluation [15]. Since the sensitivity of pleural fluid cytology is only approximately 60% [31], invasive procedures, such as image-guided biopsy, thoracoscopy, and surgery, are often required in patients with cytologically inclusive or benign reports and localized chest lesions. With limited data from our subgroup analysis, DWI showed great potential in the diagnosis of patients without N3 lymph node or extra-thoracic lesions. Including this modality in the diagnosis strategy reduced the number of unnecessary invasive procedures required. Whether DWI could be used in these patients needs to be explored in future prospective trials with larger sample sizes.

One major limitation of this study is the relatively small sample size, especially the small number of patients classified as benign. However, the targeted population was defined as having suspected PM, so most patients with benign effusion were excluded in this cohort. Another limitation is that positron emission tomography (PET) was not assessed in this study. However, several studies have shown that PET should not be routinely recommended for the diagnosis of pleural effusion [14], and the increased cost and use of ionizing radiation limit its application. In contrast, the relatively low cost and absence of radioactive contamination strengthen the appeal of DWI for clinical use.

In conclusion, this study showed that DWI is a useful tool to differentiate malignant from benign pleural lesions. To our knowledge, this is the only study to include this modality in the diagnostic algorithm of suspected PM. This approach effectively avoided unnecessary invasive procedures in patients without N3 lymph node and extra-thoracic lesions. Further prospective large-sample studies should investigate whether DWI could be applied in the preoperative evaluation of patients with potentially resectable thoracic tumors.

## Supplementary information

ESM 1 (DOCX 155 kb)

## Abbreviations

ADC: Apparent diffusion coefficient
DWI: Diffusion-weighted imaging
MRI: Magnetic resonance imaging
NPV: Negative predictive value
PET: Positron emission tomography
PM: Pleural malignancy
PPV: Positive predictive value
ROI: Region of interest
SCC: Squamous cell carcinoma
SCLC: Small cell lung cancer.

## Declarations

### Guarantor

The scientific guarantor of this publication is Jian Zhang.

### Statistics and biometry

No complex statistical methods were necessary for this paper.

### Study subjects or cohorts overlap

Some study subjects or cohorts have been previously reported in “DW MRI at 3.0 T versus FDG PET/CT for detection of malignant pulmonary tumors”.

### Methodology

• retrospective

• diagnostic study

• performed at one institution
