# Supplementary material for: Diffusion-weighted imaging diagnostic algorithm in patients with suspected pleural malignancy
Source: Eur Radiol. 2021 May 28;31(12):9038–47. doi: 10.1007/s00330-021-08013-6 (PMC8589770; doi:10.1007/s00330-021-08013-6)

Supplemental Table 1. Scoring system of CT^s^.

| CT^s^ features | Score | Benign  (n = 18) | Malignant  (n = 52) | P |
| --- | --- | --- | --- | --- |
| Any pleural lesion ≥1 cm (nodule, mass, thickening) | 5 | 5 (27.8%) | 26 (50.0%) | 0.102 |
| Lung mass or lung nodules ≥1 cm | 3 | 9 (50.0%) | 49 (94.2%) | 1.07 × 10^4^ |
| Liver metastasis | 3 | 0 (0.0%) | 1 (1.9%) | 1.000 |
| Abdominal mass | 2 | 0 (0.0%) | 1 (1.9%) | 1.000 |
| Absence of pleural loculation | 2 | 4 (22.2%) | 22 (42.3%) | 0.129 |
| No pericardial effusion | 2 | 15 (83.3%) | 43 (82.7%) | 1.000 |
| Non-enlarged cardiac silhouette | 2 | 12 (66.7%) | 39 (75.0%) | 0.545 |

For each patient, sum scores of CT^s^ features ≥7 were considered pleural malignancy.[^13^](#_ENREF_13)

Supplemental Table 2. Visual features of PM on CT.

| CT signs | Benign  (N = 18) | Malignant  (N = 52) | P |
| --- | --- | --- | --- |
| Circumferential pleural thickening | 1 (5.6%) | 3 (5.8%) | 1.000 |
| Nodular pleural thickening | 7 (38.9%) | 32 (61.5%) | 0.095 |
| Partial pleural thickening >1 cm | 0 (0.0%) | 1 (1.9%) | 1.000 |
| Mediastinal pleural involvement | 0 (0.0%) | 2 (3.8%) | 1.000 |
| Chest wall invasion and rib or centrum destruction at multiple sites | 1 (5.6%) | 2 (3.8%) | 1.000 |
| Nodular pleural thickening and mediastinal pleural involvement | 0 (0.0%) | 1 (1.9%) | 1.000 |
| Nodular pleural thickening and chest wall invasion and rib or centrum destruction at multiple sites | 0 (0.0%) | 1 (1.9%) | 1.000 |
| Pleural effusion only | 9 (50.0%) | 14 (26.9%) | 0.041 |

PM: pleural malignancy; CT: computed tomography.

Supplemental Table 3. Sum scoring results of CT^2^.

| Sum score | Pleural benign (N = 18) | Pleural malignant (N = 52) |
| --- | --- | --- |
| 2 | 5 (27.8%) | 0 (0.0%) |
| 3 | 0 (0.0%) | 3 (5.8%) |
| 4 | 1 (5.6%) | 1 (1.9%) |
| 5 | 4 (22.2%) | 5 (9.6%) |
| 6 | 1 (5.6%) | 0 (0.0%) |
| 7 | 1 (5.6%) | 11 (21.2%) |
| 8 | 0 (0.0%) | 2 (3.8%) |
| 9 | 2 (11.1%) | 4 (7.6%) |
| 10 | 1 (5.6%) | 2 (3.8%) |
| 11 | 1 (5.6%) | 3 (5.8%) |
| 12 | 2 (11.1%) | 10 (19.2%) |
| 13 | 0 (0.0%) | 1 (1.9%) |
| 14 | 0 (0.0%) | 10 (19.2%) |

CT: computed tomography.

Supplemental Table 4. Visual features of PM on DWI.

| DWI | Benign  (N = 18) | Malignant  (N = 52) | P |
| --- | --- | --- | --- |
| Hyperintense pleura on DWI | 9 (50.0%) | 49 (94.2%) | 1.07 × 10^−4^ |
| Circumferential pleural thickening | 5 (27.8%) | 20 (38.5%) | 0.415 |
| Nodular pleural thickening | 3 (16.7%) | 21 (40.4%) | 0.068 |
| Thready pleural thickening | 1 (5.6%) | 2 (3.8%) | 1.000 |
| Mediastinal pleural involvement | 0 (0.0%) | 6 (11.5%) | 0.327 |
| Chest wall invasion and rib or centrum destruction at multiple sites | 0 (0.0%) | 8 (15.4%) | 0.103 |
| Circumferential pleural thickening and mediastinal pleural involvement | 0 (0.0%) | 5 (9.6%) | 0.318 |
| Circumferential pleural thickening and chest wall invasion and rib or centrum destruction at multiple sites | 0 (0.0%) | 1 (1.9%) | 1.000 |
| Nodular pleural thickening and chest wall invasion and rib or centrum destruction at multiple sites | 0 (0.0%) | 1 (1.9%) | 1.000 |
| Thready pleural thickening and chest wall invasion and rib or centrum destruction at multiple sites | 0 (0.0%) | 1 (1.9%) | 1.000 |
| No signal on DWI | 9 (50.0%) | 3 (5.8%) | 1.07 × 10^−4^ |

PM: pleural malignancy; DWI: diffusion-weighted imaging.

Supplemental Table 5. Qualitative assessment of DWI and CT for the diagnosis of PM in patients without N3 lymph node or extra-thoracic lesions.

|  | Sensitivity (%)  (95% CI) | Specificity (%)  (95% CI) | PPV (%)  (95% CI) | NPV (%)  (95% CI) | Accuracy (%)  (95% CI) |
| --- | --- | --- | --- | --- | --- |
| DWI | 100.0 (13/13)  (75.3–100.0) | 86.7 (13/15)  (59.5–98.3) | 86.7 (13/15)  (59.5–98.3) | 100.0 (13/13)  (75.3–100.0) | 92.9 (26/28)  (76.5–99.1) |
| CT | 76.9 (10/13)  (46.2–95.0) | 66.7 (10/15)  (38.4–88.2) | 66.7 (10/15)  (38.4–88.2) | 76.9 (10/13)  (46.2–95.0) | 71.4 (20/28)  (51.3–86.8) |

DWI: diffusion-weighted imaging; CT: computed tomography; PM: pleural malignancy; CI: confidence interval.

Supplemental Figure 1. ADC comparison between the malignant and benign groups. Fifty patients in the malignant group and 11 patients in the benign group were analyzable. The average ADC in the malignant group was 1.15 ± 0.32 × 10^−3^ mm^2^/sec, and the average ADC in the benign group was 1.46 ± 0.68 × 10^−3^ mm^2^/sec. There was no statistically significant difference between groups (P *=* 0.161). ADC: apparent diffusion coefficient.


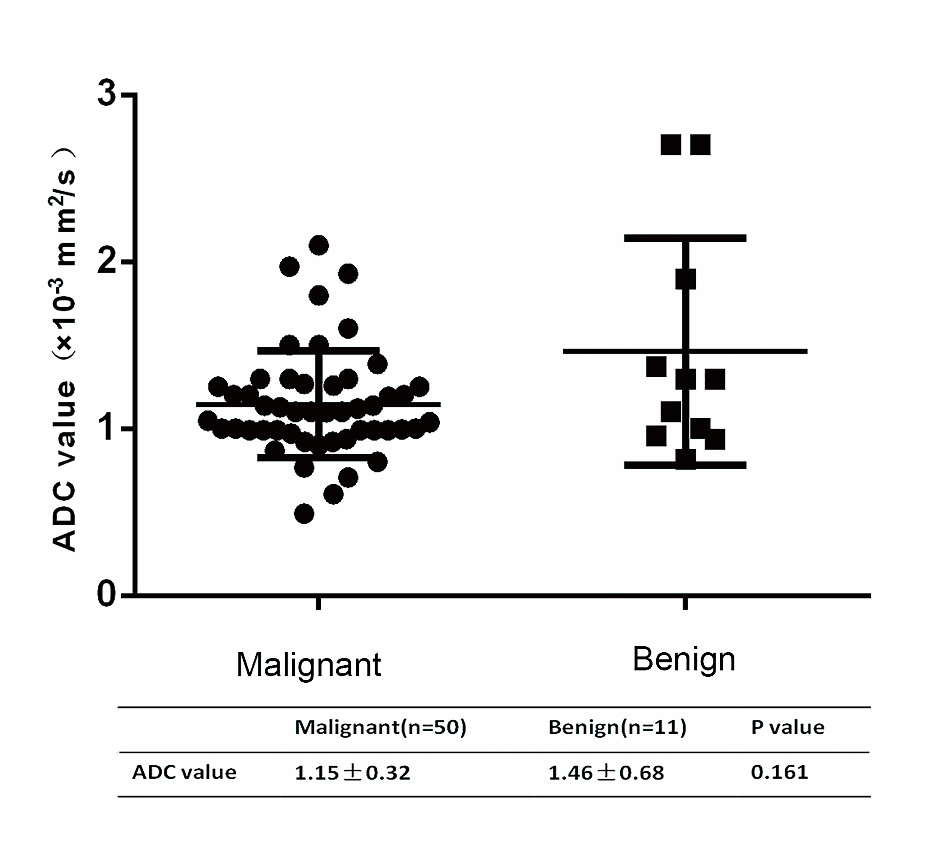

Supplement: Supplementary file 1 — (DOCX 155 kb) [file 330_2021_8013_MOESM1_ESM.docx]
